# CutLang v2: Advances in a Runtime-Interpreted Analysis Description Language for HEP Data

**DOI:** 10.3389/fdata.2021.659986

**Published:** 2021-06-07

**Authors:** G. Unel, S. Sekmen, A. M. Toon, B. Gokturk, B. Orgen, A. Paul, N. Ravel, J. Setpal

**Affiliations:** ^1^ Department of Physics and Astronomy, University of California, Irvine, Irvine, CA, United States; ^2^ Department of Physics, Kyungpook National University, Daegu, South Korea; ^3^ Department of Computer Software Engineering, Saint Joseph University of Beirut, Beirut, Lebanon; ^4^ Department of Physics, Bogazici University, Istanbul, Turkey; ^5^ The Abdus Salam International Centre for Theoretical Physics, Trieste, Italy; ^6^ Department of Physics, University of Ankatso, Antananarivo, Madagascar; ^7^ R.N. Podar School, Mumbai, India

**Keywords:** LHC, collider, run time analysis, analysis description language, CutLang

## Abstract

We will present the latest developments in CutLang, the runtime interpreter of a recently-developed analysis description language (ADL) for collider data analysis. ADL is a domain-specific, declarative language that describes the contents of an analysis in a standard and unambiguous way, independent of any computing framework. In ADL, analyses are written in human-readable plain text files, separating object, variable and event selection definitions in blocks, with a syntax that includes mathematical and logical operations, comparison and optimisation operators, reducers, four-vector algebra and commonly used functions. Adopting ADLs would bring numerous benefits to the LHC experimental and phenomenological communities, ranging from analysis preservation beyond the lifetimes of experiments or analysis software to facilitating the abstraction, design, visualization, validation, combination, reproduction, interpretation and overall communication of the analysis contents. Since their initial release, ADL and CutLang have been used for implementing and running numerous LHC analyses. In this process, the original syntax from CutLang v1 has been modified for better ADL compatibility, and the interpreter has been adapted to work with that syntax, resulting in the current release v2. Furthermore, CutLang has been enhanced to handle object combinatorics, to include tables and weights, to save events at any analysis stage, to benefit from multi-core/multi-CPU hardware among other improvements. In this contribution, these and other enhancements are discussed in details. In addition, real life examples from LHC analyses are presented together with a user manual.

## 1 Introduction: Domain Specific Languages for High Energy Physics Analysis

High energy physics (HEP) collider data analyses nowadays are performed using complex software frameworks that integrate a diverse set of operations from data access to event selection, from histogramming to statistical analysis. Mastering these frameworks requires a high level knowledge of general purpose languages and software architecture. Such requirements erect a barrier between data and the physicist who may simply wish to try an analysis idea. Moreover, even for experienced physicists, obtaining a complete view of an analysis is difficult because the physics content (e.g., object definitions, event selections, background estimation methods, etc.) is often scattered throughout the different components of the framework. This makes developing, understanding, communicating and interpreting analyses very challenging. At the LHC, almost all analysis teams have their own frameworks. There are also frameworks like CheckMate (Drees et al., 2015; Kim et al., 2015; Tattersall et al., 2016) and MadAnalysis (Conte et al., 2013; Conte and Fuks, 2014) for phenomenology studies, and Rivet (Waugh et al., 2006; Buckley et al., 2013) focused on preserving LHC analyses with unfolded results for comparison with Monte Carlo event generator predictions. Yet, working with multiple frameworks is an extra challenge, since each framework has a different way of implementing the physics content.

It is therefore crucial to invest time in alternative approaches aiming towards the rather elusive point of easy to learn, expressive, extensible, and effective analysis ecosystem that would allow to shift the focus away from programming technicalities to physics analysis design. One way to achieve this is via a well-constructed set of libraries in a GPL supplemented with a well-designed interfaces that intrinsically imply a standard and user-friendly analysis structure. A most promising example in this area is the Scientific Python ecosystem SciPy^1^ which brings together a popular GPL and a rich collection of already existing bricks of classic numerical methods, plotting and data processing tools. Frameworks can be built based on the SciPy ecosystem for effective analysis, such as Coffea framework (Gray et al., 2020) that provides a user interface for columnar analysis of HEP data.

The approach that we propose in this paper to address these difficulties is the consideration of a domain specific language (DSL) capable of describing the analysis flow in a standard and unambiguous way. A DSL could be based on a completely original syntax, or it could be based on the syntax of a general purpose language, such as Python. The important aspect would be to provide a unique and organized way of expressing the analysis content. Applying the DSL concept to HEP analysis was first thoroughly explored as a community initiative by a group of experimentalists and phenomenologists in the 2015 Les Houches PhysTeV workshop led to the initial design of LHADA (Les Houches Analysis Description Accord), to systematically document and run the contents of LHC physics analyses (Brooijmans et al., 2016; Brooijmans et al., 2018; Brooijmans et al., 2020). At the same time, some of the LHADA designers were already developing CutLang (Sekmen and Unel, 2018; Unel et al., 2019), an interpreted language directly executable on events. Being based on the same principles, in 2019, LHADA and CutLang were merged by combining the best ideas from both into a unified DSL called “Analysis Description Language (ADL)” (Prosper et al., 2020), which is described in this paper.

While the prototyping of LHADA, CutLang and ADL was in progress, parallel efforts arose in the LHC community with the aim to improve and systematize analysis development infrastructures. One approach views each event as a database that can be queried using a language inspired by SQL, and has been prototyped in LINQtoROOT (Gordon, 2010) and FemtoCode (Pivarski, 2006a). The SQL-like model is being further explored in hep_tables and dataframe_expressions (Watts, 2020) that work together to allow easy columnar-like access to hierarchical data, and in the recent experimental language PartiQL (Pivarski, 2006b) designed to inject new ideas into DSL development and its extension AwkwardQL (Gray, 2020), designed to perform set operations on data expressed as awkward arrays. Another study explored building a DSL embedded within YAML to describe and manage analysis content such as definitions, event selection, histogramming as well as perform data processing. The YAML-based language was integrated into the generic Python framework F.A.S.T. (Krikler, 2020).

The focused DSL developments for analyses are relatively new, but a DSL has been long embedded within the ROOT framework (Brun and Rademakers, 1997) under the guise of TTreeFormula, TTree::Draw and TTree::Scan, which allow visual or textual representation of TTree contents for simple and quick exploratory analysis This DSL is however limited only to simple arithmetic operations, mathematical functions and basic selection criteria. Recently, ROOT developers introduced RDataFrame, a tool to process and analyze columnar datasets as a modern alternative for data analysis (Piparo et al., 2019). Although RDataFrame is not a DSL itself, it implements declarative analysis by using keywords for transformations (e.g., filtering data, defining new variables) and actions (e.g., creating histograms), and is interfaced to the ROOT classes TTreeReader and TTreeDraw. RDataFrame recently led to the development of the preliminary version of another DSL and its interpreter called NAIL (Natural Analysis Implementation Language) (Rizzi, 2020a). NAIL, written in Python. It takes CMS NanoAOD (Rizzi, 2020b) as an input event format and generates RDataFrame-based C++ code, either as a C++ program or as a C++ library loadable with ROOT.

All these different approaches and developments were discussed among experimentalists, phenomenologists and computer scientists in the first dedicated workshop “Analysis Description Languages for the LHC” at Fermilab, in May 2019.^2^ The workshop resulted in an overall agreement on the potential usefulness of DSLs for HEP analysis, elements of a DSL scope and an inclination to pursue multiple alternatives with the ultimate goal of a common DSL for the LHC that combines the best elements of the different approaches (Sekmen et al., 2020). The activities in DSL development are therefore ongoing with a fast pace.

This initial positive feedback has motivated further progress in ADL, which will be described here. ADL is a declarative language that can express the mathematical and logical algorithm of a physics analysis in a human-readable and standalone way, independent of any computing frameworks. Being declarative, ADL expresses the analysis logic without explicitly coding the control flow, and is designed to describe what needs to be done, but not how to do it. This consequently leads to a more tidy and efficient expression and eliminates programming errors. At its current state, ADL is capable of describing many standard operations in LHC analyses. However, it is being continuously improved and generalized to address an even wider range of analysis operations.

ADL is designed as a language that can be executed on data and used in real life data analyses. An analysis written with ADL could be executed by any computing framework that is capable of parsing and interpreting ADL, hence satisfying the framework independence. Currently, two approaches have been studied to realize this purpose. One is the transpiler approach, where ADL is first converted into a general purpose language, which is in turn compiled into code executable on events. A transpiler called adl2tnm converting ADL to C++ code is currently under development (Brooijmans et al., 2018). Earlier prototype transpilers converting LHADA into code snippets that could be integrated within CheckMate (Drees et al., 2015; Kim et al., 2015; Tattersall et al., 2016) and Rivet (Waugh et al., 2006; Buckley et al., 2013) frameworks were also studied. The other approach is that of runtime interpretation. Here ADL is directly executed on events without being intermediately converted into a code requiring compilation. This approach was used for developing CutLang (Sekmen and Unel, 2018; Unel et al., 2019).

In this paper, we focus on CutLang and present in detail its current state denoted as CutLang v2, which was achieved after many improvements on the early prototype CutLang v1 introduced in (Sekmen and Unel, 2018). Hereafter, CutLang v2 will be referred to as CutLang for brevity. The main text emphasizes the novelties that led to ADL and improved CutLang. We start with an overview of ADL in Section 2, then proceed with describing technicalities of runtime interpretation with CutLang in Section 3. We next present the ADL file structure and analysis components that can be expressed by ADL, focusing on the new developments and recently added functionalities in Section 4. This is followed by Section 5 describing analysis output, again focusing on new additions, Section 6, explaining the newly-added multi-threaded run functionality, Section 7 on CutLang code maintenance and recently incorporated continuous integration, Section 8 detailing studies on analyses implementation, and conclusions in Section 9. The full description of the current language syntax is given in the form of a user manual in Supplementary Appendix A, followed by a note on the CutLang framework and external user functions in Supplementary Appendix B.

## 2 Analysis Description Language Overview: File and Functions

In ADL, the description of the analysis flow is done in a plain, easy-to-read text file, using syntax rules that include standard mathematical and logical operations and 4-vector algebra. In this ADL file, object, variable, event selection definitions are clearly separated into blocks with a keyword value/expression structure, where keywords specify analysis concepts and operations. Syntax includes mathematical and logical operations, comparison and optimization operators, reducers, 4-vector algebra and HEP-specific functions (e.g., dϕ, dR). However, an analysis may contain variables with complex algorithms non-trivial to express with the ADL syntax [e.g., $M_{T2}$ (Barr et al., 2003), aplanarity] or non-analytic variables (e.g., efficiency tables, machine learning discriminators). Such variables are encapsulated in self-contained, standalone functions which accompany the ADL file. Variables defined by these functions are referred to from within the ADL file. As a generic rule, all keywords, operators and function names are case-insensitive. n The language content, syntax rules, and working examples of self-contained functions will be presented in the coming sections, after a technical introduction of the CutLang interpreter.

## 3 Technical Background of the CutLang Interpreter

An interpreted analysis system makes adding new event selection criteria, changing the execution order or cancelling analysis steps more practical. Therefore CutLang was designed to function as a runtime interpreter and bypass the inherent inefficiency of the modify-compile-run cycle. Avoiding the integration of the analysis description in the framework code also brings the huge advantage of being able to run many alternative analysis ideas in parallel, without having to make any code changes, hence making the analysis design phase more flexible compared to the conventional compiled framework approach.

**FIGURE 1:**
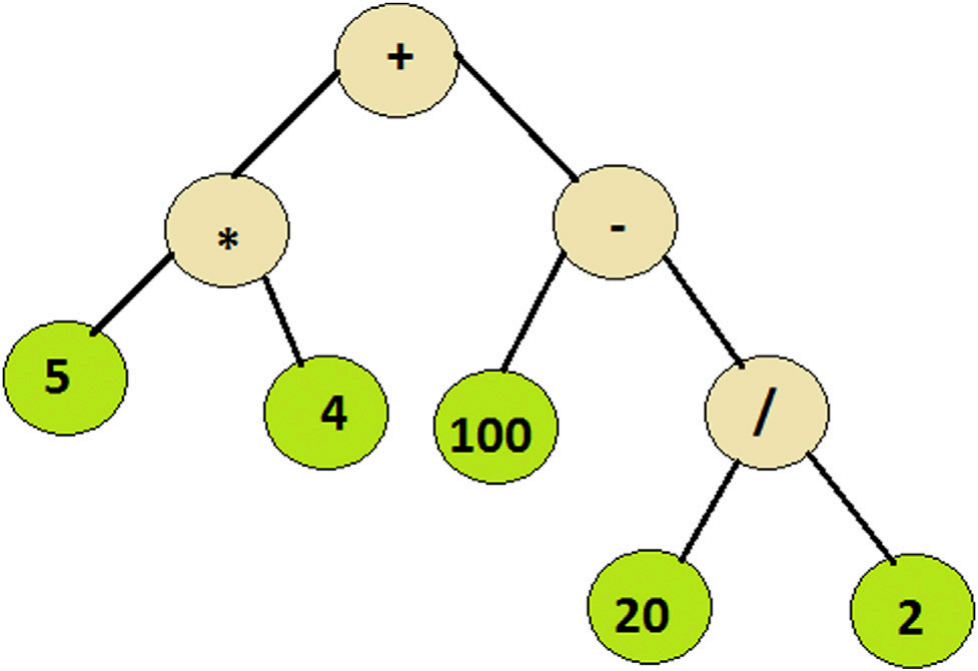
An expression tree example: the program traverses the tree from right to left evaluating the encountered functions from bottom to top.

CutLang runtime interpreter is written in C++, around function pointer trees representing different operations such as event selection or histogramming. Therefore processing an event with a cutflow table becomes equivalent to traversing multiple expression trees with arbitrary complexities, such as the one shown in Figure 1. Here physics objects are given as arguments.

Handling of the Lorentz vector operations, pseudo-random number generation, input-output file and histogram manipulations are all based on classes of the ROOT data analysis framework (Brun and Rademakers, 1997). The actual parsing of the ADL text relies on automatically generated dictionaries and grammar based on traditional Unix tools, namely, Lex and Yacc.^3^ The ADL file is split into tokens by Lex, and the hierarchical structure of the algorithm is found by Yacc. Consequently, CutLang can be compiled and operated in any modern Unix-like environment. The interpreter should be compiled only once, during the installation or when optional external functions for complex variables are added. Once the work environment is set up, the remainder is mostly a think-edit-run-observe cycle. The parsing tools also address the issue of possible user mistakes with respect to the syntax. CutLang output clearly indicates the problem, and the line number of the offending syntax error. However the logical inconsistencies, such as imposing a selection on the third jet’s momentum while only requesting at least two jets are not yet handled. Ensuring the consistency of the algorithm needs to be done by the user. Input and output to CutLang is via ROOT files. The description of the input files and event formats are given below while the description of the output file and its contents are given in Section 5.

### 3.1 Event Input

The CutLang framework takes the input event information in the ROOT ntuple format and can work with different input event data types each implemented as a plug-in. Widely used event formats such as ATLAS and CMS open data,^4^ CMS NanoAOD (Rizzi, 2020b), Delphes (de Favereau et al., 2014) and LHCO event are by default recognized and can be directly used. New or custom input event formats can also be easily added via usage of event class headers via a well-defined procedure described in Supplementary Appendix B.3. The potential changes in the existing event formats and addition of new event formats currently need to be adapted manually following the mentioned procedure. CutLang has its own internal event format called LVL0. The contents of the input event formats including all particle types and event properties are worked through an internal abstraction layer and adapted to LVL0, which, in turn connects to the syntax of ADL. The purpose of this approach is to have ADL be independent of the input file format and be capable of running the same ADL analysis with CutLang on any input file. This implies that only a subset of event content is readily recognized via CutLang when expressed within the ADL syntax. However, any event variables or attributes included in the existing event files and formats can be easily added through external user functions. This way, they can be referred to within the ADL files and be recognized by CutLang. The practical details of this procedure can be found in Supplementary Appendix B.2.

## 4 Description of the Analysis Contents

We will now explain in detail which analysis components and physics algorithms can be described by ADL and processes with CutLang. We will prioritize highlighting the many novelties added and improvements that took place since the original versions CutLang v1 and LHADA. The descriptions here concentrates on the concepts and content that can be expressed and processed by ADL and functionalities of CutLang v2, rather than attempting to give a full layout of syntax rules, which is independently provided in the user manual in Supplementary Appendix A.

### 4.1 Analysis Description Language File Structure in CutLang

As a runtime interpreter, CutLang processes events in a well-defined order. It executes the commands in the ADL file from top to bottom. Therefore, the ADL files are required to describe the analysis flow in a certain order. Some dedicated execution commands are also used within the ADL file, in order to facilitate the runtime interpretation. Throughout the ADL file, the mass, energy and momentum are all written in Giga Electron Volt (GEV) and angles in radians. User comments and explanations should be preceded by a hash (#) sign. To be executable with CutLang, an ADL file would consist of five possible sections described below, out of which, existence of one section is mandatory:

**initializations:** This section contains commands that are related to analysis initialization and set up, for which, the relevant keywords are summarized in Table 1. The keywords and values are separated by an equal sign. The last two lines in the table refer to the lepton (electron or muon) triggers. Their utilization is described in Supplementary Appendix A.2, it is worth noting at this point that Monte Carlo (MC) simulation weights are not taken into account when the trigger value is set to data.
**countformats:** This section is used for setting up the recording of already existing event counts and errors, e.g., from an experimental paper publication. It is therefore not directly relevant for event processing, but rather for studying the interplay between the results of the current analysis and its published experimental counterpart. More generally, it is used to express any set of pre-existing counts of various signals, backgrounds, and data (together with their error) of an analysis.
**definitions1:** This section is used for defining aliases for objects and variables, in order to render them more easily referable and readable in the rest of the analysis description. For example, it can introduce shortcuts like Zhreco for a hadronically reconstructed Z boson, or values like mH, i.e., mass of a reconstructed Higgs boson. These definitions can only be based on the predefined keywords and objects.
**objects:** This section can be used to define new objects based on predefined physics objects and shorthand notations declared in definitions1.
**definitions2:** This section is allocated for further alias or shorthand definitions. Definitions here can be based on objects in the previous section and predefined particles.
**event categorization:** This section is used for defining event selection regions and criteria in each region. Running with CutLang requires having at least one selection region with at least one command, which may include either a selection criterion or a special instruction to include MC weight factors or to fill histograms.

**TABLE 1:** Initialization keywords and their possible values.

Keyword	Explanation
SkipHistos	Skip (=1) or Display (=0) the histograms in final efficiency table
SkipEffs	Skip (=1) or Display (=0) the final efficiency table
TRGm	0=Off, 1=Data, 2=MC for muons
TRGe	0=Off, 1=Data, 2=MC for electrons
RandomSeed	random number generator seed, an integer

We next describe the detailed contents and usage of these sections.

### 4.2 Object Definitions

Generally, the starting point in an analysis algorithm is defining and selecting the collections of objects, such as jets, *b* jets, electrons, muons, taus, missing transverse energy, etc. that will be used in the next steps of the analysis. Usually, the input events contain very generic and loose collections for objects, which need to be further refined for analysis needs. CutLang is capable of performing a large variety of operations on objects, including deriving new objects via selection, combining objects to reconstruct new objects, accessing the daughters and constituents of objects. Once an object is defined, it is also possible to find objects with a minimum and maximum of a given attribute within the object’s collection, or sort the collection according to an attribute.

In the ADL notation, object collection definitions are clearly separated from the other analysis tasks. Here the term object is used interchangeably with object collection. Each object is defined within an individual object block uniquely identified with the object’s name. These blocks, starting with the input object collection(s)’s name(s), list different types of operations afterwards.

CutLang automatically retrieves all standard object collections from the input event file without the need for any explicit user statements within the ADL file. It can read events with different formats, such as Delphes fast simulation (de Favereau et al., 2014) output, CMS NanoAOD (Rizzi, 2020b), ATLAS or CMS open data^4^ ntuples and recognize the object collections in these. One property unique to CutLang is that it is designed to map input object collections to common, standard object names with a standard set of attributes, as described in Supplementary Appendices A.2 and A.3. For example, AK4jets collection in CMSNanoAOD and JET collection in Delphes are both mapped to Jet. This approach allows to process the same ADL file on different input event formats, and has proven very useful in several simple practical applications. However, we also recognize that this approach only works when different input collections have matching properties, e.g., when Delphes electrons and CMS electrons have to the same identification criteria which can be mapped to the same identification attribute, or a Delphes jet and an ATLAS jet use the same b-tagging algorithm that can be mapped to the same b-tagging attribute. Therefore, other interpreters of ADL may choose to use input collection and attribute names as they are, in order to be more unambiguous. Allowing to practice different approaches with advantages for different use cases, while still adhering to the principle of clarity is a significant aspect of ADL.

The most common object operation is to take the input object collection and filter a subset by applying a set of selection criteria on object attributes. This can be done very straightforwardly in ADL by listing each selection criterion in consecutive lines. The objects in the input collection satisfying the criteria can be either selected or rejected using the select or reject keywords. Comparison operators such as =, != >, <, >=, <= can directly be used for expressing the criteria. Logical operators AND, OR and NOT can be used for expressing composite or reverted criteria. A complete list of these operators can be found in Supplementary Appendix A.5.

It is also possible to filter an object collection based on other object collections, such as in the cases of object cleaning or matching. For example, one can reject jets overlapping with photons, or select boosted W jets matching generator level W bosons. Such operations involve intrinsic loops, which are readily handled by CutLang. Functions such as δϕ or angular distance δR can be readily used when comparing objects. Given an initial object collection, one can consecutively derive several objects. For example jets can be filtered to obtain cleanjets, while cleanjets can be further filtered to obtain verycleanjets. One can also use the same initial collection to define different collections such as taking muons and imposing different criteria to obtain loosemuons and tightmuons.

Another very common operation is to combine objects to reconstruct new objects, such as combining two leptons to form a *Z* boson. Sometimes, the reconstruction could be very straightforward, as in requesting to reconstruct only a single *Z* boson per each event. However, in other cases, one might have to reconstruct as many *Z* bosons as possible. In each case, reconstructed candidates might undergo filtering or selection of a single most optimal candidate among all candidates. Combination operations are very diverse, and finding a completely generic expression for them is non-trivial. In its v1, CutLang could reconstruct an explicitly defined number of objects per event. It could find the object satisfying given criteria by performing optimization operations. In v2, CutLang has been generalized to reconstruct any number of objects, by taking into account the combinatorics. Selection criteria can also be imposed on both the input and reconstructed objects. Technical information on how to perform combinations is provided in Supplementary Appendix A.9.3.

Another common situation is when objects in a collection are individually associated to other collections. Examples include mothers or daughters of generator level particles, subjets or constituents of jets, associated tracks of leptons or jets. As a first CutLang was adapted in v2 to work with jet constituents using the syntax described in Supplementary Appendix A.9.7. Another example of association is daughters of generator truth level particles. If an analysis if performed directly on generator level particles, or if a study is required on truth level particles, information such as PDGID codes or decay chain become relevant. CutLang is now capable of accessing PDGID and the decay products of a particle (referred as “daughters” in HEP), with the syntax described in Supplementary Appendices A.3.1 and A.9.8. CutLang provides both the number of daughters and a modifier to refer to the daughters. Work is in progress for finding a generalizable syntax for object association expressions.

Members of object collections can be directly accessed via their indices. Being declarative, ADL syntax does not include explicit statements for looping over object collections, and CutLang is capable of interpreting this implicit looping. For example, when filtering a jet collection, one might apply a cleaning criterion which requires no electron to be in the proximity of the jet defined by a radius. Applying this criterion requires looping over electrons, however it suffices to write the electron object’s name in order for CutLang to interpret implicit looping based on the context. In other cases, it might be necessary to access only a subset of the collection, such as when imposing a selection on the δϕ between first 3 jets with highest $p_{T}$ and the missing transverse momentum. ADL and CutLang were updated to allow such operations. The Python slice notation has been adapted for expressing subset ranges in object collections, as described in Supplementary Appendix A.9.4.

Input or defined object collections are by default sorted by CutLang in the order of decreasing transverse momentum $p_{T}$. ADL can express sorting object collections according to any feature, in ascending or descending order, and CutLang is capable of performing such sorting operations. Moreover, so-called “reducers” can be applied for extracting values from existing object collections. One case is the capability to extract the maximum or minimum value of a given attribute in an object collection. For example, CutLang can give the maximum $p_{T}$ possessed by a jet in a jet collection, or minimum value of isolation possessed by an electron in an electron collection. Another case is the summation operation, where one can sum over the values of a given attribute over the whole collection. The most common use case here is the summation of object $p_{T}$s to obtain event variables such as the hadronic transverse energy $H_{T}$. Sorting and reducers are recent additions to ADL and CutLang and the details on their implementation and usage are given in Supplementary Appendices A.9.2, A.9.5, A.9.6 and in the examples referred to in Section 8.

### 4.3 Object or Event Variables

An object variable is a quantity defined once per object, such as a jet’s transverse momentum $p_{T}$ or an electron’s relative isolation. An event variable is a quantity defined once per event, such as missing transverse energy $E_{T}^{miss}$, number of electrons selected using the tight criteria, $p_{T}$ of the highest $p_{T}$ jet, transverse mass calculated using the highest $p_{T}$ lepton and $E_{T}^{miss}$. Object and event variables used in object definitions or event categorization in an analysis are not always fully provided in the input event data. These quantities therefore need to be computed during the analysis using the existing inputs. ADL is designed to allow definition of such new variables in two ways. Simple variables that could be described analytically using a single line formula can be expressed within the ADL file using mathematical operations. A classic example would be that of the definition of transverse mass obtained from a visible object, such as a lepton, and the missing transverse energy. To enable writing these simple formulas, CutLang is capable of parsing and processing operators such as $+,\;-,\;\text{*},\;/,\;\hat{\$}\;$. CutLang has also incorporated a series of internal functions to express other operations such as abs(), sqrt(), sin(), cos(), tan() and log(). Reducer operators used for reducing collections to a single value, e.g., size(), sum(), min(), max() are also available for computing quantities. For example, the hadronic transverse momentum $H_{T}$ can be computed from all jets in an event using the sum() reducer as sum(pT(jets)).

However, in many cases, variables are defined by complex algorithms non-trivial to express. Examples such as angular separation dR, aplanarity, stransverse mass $M_{T2}$ (Barr et al., 2003), razor variables (Rogan, 2010), etc. either cannot be easily written using the available operators or require multiple steps of calculation. Some of these algorithms, like angular separation and razor variables were predefined as internal functions in CutLang, and more, like $H_{T}$ and $M_{T2}$ were added recently. A list of existing variables can be found in Supplementary Appendix A.3. Other algorithms can be easily incorporated by the user following the recently generalized recipe in Supplementary Appendix B. Another class of sophisticated variables include quantities defined from numerical functions, such as object or trigger efficiencies used to compute object or event weights, provided in tables or histograms, or discriminators/efficiencies computed via machine learning models. All these variables are incorporated by being defined in independent, self-encapsulated functions outside the ADL file and referring to them within the ADL file. These external user functions should be seen as a natural extension of the language. The ultimate aim is to provide these functions in a well-defined and straightforwardly extendable database.

The expressions for variables, whether they are built directly using the available mathematical operators or indirectly via internal or user functions, can be written openly in the place of usage, e.g., in the line when a selection is applied on the variable. Alternatively, if the variable is used multiple times in an analysis, e.g., in different selection regions, it can be defined once, using the define keyword, which allows to assign an alias name to the variable. Currently, defining aliases using the define keyword is only possible for event variables in CutLang, but not for object variables. In CutLang, the define expressions are uniquely placed at the end of the object blocks and before the beginning of the event selection.

### 4.4 Event Categorization

In a typical collider analysis, events are categorized based on different sets of selection criteria applied on event variables into a multitude of signal regions enhancing the presence of the signal of interest, or control or validation regions used for estimating backgrounds. These regions can be derived from each other, and can be correlated or uncorrelated depending on the case. ADL organizes event categorization by defining each selection region in an independent region block^5^ and labels each region with a unique name. The region blocks mainly consist of a list of selection criteria. As in the case for objects, each criterion is stated in a line starting with a select or a reject keyword, which allows to select or reject the events satisfying the criterion, respectively. Comparison operators, logical operators and ternary operator, syntax for which is described in Supplementary Appendix A.5 are used for expressing the criteria. Another operation that can be performed within the context of event classification is $\chi^{2}$ optimization for reconstructed quantities, whose syntax is described in Supplementary Appendix A.6. An example would be finding among several top quark candidates, the candidate with mass closest to the top quark mass, and using the optimal candidate’s properties for further selection.

ADL and CutLang allow deriving selection regions from each other, e.g., deriving multiple signal regions from a baseline selection region. This is done by simply referring to the baseline region by name in the new region’s block, and not repeating the whole selection every time.

In many analyses, especially those targeting searches for new physics, events in given search regions are partitioned into many bins based on one or more variables, e.g., $H_{T}$, $E_{T}^{miss}$ or some invariant mass. Data counts and background estimates in these bins constitute the result of the analysis. With the increased data, recent LHC analyses, especially inclusive searches for new physics may contain hundreds of bins. Treating each bin as an independent search region and writing a separate block for each would be highly impractical. As an alternative, recently, the capability of binning the events in a given region was added to ADL and CutLang through the bin keyword. Bins in a region, by definition, are to be non-overlapping. The CutLang interpreter and framework operate based on this principle, and skip an event once it is classified into a bin. This property distinguishes bins from regions, as different regions can be overlapping, and a given event is evaluated for all regions, independent of whether it is selected or not by the preceding regions. Bins can be described in two ways: when the binning is done using only a single variable, all bins can be defined in a single line, by specifying the variable name and the bin intervals. When bins are defined based on multiple variables, this way of description can become ambiguous, and a more explicit description, where each bin is defined in one dedicated line can be used. The usage and syntax of the bin keyword is described in Supplementary Appendix A.11.1. In case multiple regions would have the same binning (e.g., a signal region and several control regions from which the background is estimated), currently, the binning definitions must be separately specified in each region independently. We are searching for a more practical way of expression which would avoid the repetition, while keeping with the human readability principle.

### 4.5 Event Weights

In an analysis, events, especially simulated events are usually weighted in order to match the real data luminosity or to correct for detector effects. CutLang has been recently adapted to incorporate the capability of applying event weights. Event weights can be applied within the region blocks via usage of the weight keyword as described in Supplementary Appendix A.10.2. A particular event selected by two different regions can receive different weights. Event weights can be either constant numbers or functions of variables. These functions may include analytical or numerical internal or user functions. Weights based on numerical functions, such as efficiencies (e.g., trigger efficiencies) can also be applied from tables written within the ADL file, as described in Supplementary Appendix A.8. The systematic way for expressing efficiencies in tables and applying them to objects and events was incorporated recently in ADL and CutLang.

### 4.6 Applying Efficiencies to Objects and Events Using the Hit-and-Miss Method

As mentioned above, applying efficiencies to events and objects, such as trigger efficiencies or object reconstruction, identification and isolation efficiencies is a common part of many analyses. Section 4.5 described how to apply the effect of event efficiencies as event weights. There is, however, another approach, which involves emulating the effects of efficiencies. This approach involves randomly accepting events or objects having a certain property, such that the total selected percentage reflects that of the efficiency. For example, if the overall reconstruction and identification efficiency for an electron with $20<p_{T}<40$ GeV and |η|<2.1 is 60%, a given MC truth electron in that $p_{T}$ and |η| range is allowed to pass the selection only with a 0.6 probability. The decision for selection is made by sampling a uniform random number between 0 and 1, and accepting the event or object if the uniform random number is greater than the efficiency value. Usually, the uncertainty on the efficiency is also taken into account when making the pass/fail decision. This is called the hit-and-miss method.

Emulating efficiencies using the hit-and-miss method is regularly used in parameterized fast simulation frameworks. It is also becoming increasingly relevant to incorporate this functionality in the analysis step, especially for the benefit of phenomenological studies targeting interpretation or testing new analysis ideas. These studies generally use events produced by fast simulation or even at truth level instead of real collision data events or MC events produced by full detector simulation as used in experimental analyses. Experimental analyses use complicated object identification criteria, which cannot be implemented by fast simulation. Moreover, it is common to see different analyses working with different identification methods for a given object (e.g., cut-based identification versus multivariate analysis-based identification for electrons), as different methods may perform better for different physics cases. Consequently, working with different phenomenology analyses each using different identification criteria requires implementing all these criteria in the simulation step, which is highly impractical. Therefore, it is helpful for the infrastructure handling the analysis step to have the capability to emulate using efficiencies.

Emulating efficiencies with uncertainties was recently incorporated in CutLang. The hit-and-miss method is applied via the internal function applyHM. In the current implementation, the efficiency values and errors versus object properties are input via table blocks in the ADL file. This will be generalized to reading efficiencies from other formats, e.g., input histograms or numerical external functions in the near future.

The applyHM function uses a uniform distribution to decide if the central value was hit (below the value) or missed (above the value), the central value itself is recalculated in case the table contains errors. The new value is recalculated each time based on a double Gaussian function with positive and negative widths which are the errors of the associated bin in the efficiency table:

$$dg\left(x\right)\equiv \sqrt{\frac{2}{\pi *\epsilon_{u}\text{*}\epsilon_{d}}}\text{*}\left[e^{-}\frac{{\left(x-\mu \right)}^{2}}{2\text{*}\epsilon_{d}^{2}}\times \theta \left(\mu \right)+e^{-}\frac{{\left(x-\mu \right)}^{2}}{2\text{*}\epsilon_{u}^{2}}\right],$$ (1)

where *μ* is the central value of the relevant bin from efficiency table, $\epsilon_{u}$ and $\epsilon_{d}$ are the errors in the same bin and finally *θ* is the unit step function. The applyHM function can both be used in the object blocks for defining derived object collections. It can also be used in the region blocks to apply efficiencies on a particular object, e.g., to check whether the jet with the highest $p_{T}$ is a b-tagged jet or not. Syntax for the applyHM function can be found in Supplementary Appendix A.9.9.

### 4.7 Histogramming

As described in the introduction, the main scope of ADL is the description of the physics content and algorithmic flow of an analysis. The language content presented up to this point serves this purpose. However further auxiliary functionalities are required for practicality while running the analysis on events. One such functionality is histogramming. Since the start of its design, CutLang has been capable of filling one-dimensional histograms of event variables. Recently, the capability of drawing two-dimensional histograms has been added. The syntax for histogramming can be found in Supplementary Appendix A.11.3. Histogramming is currently only available for event variables. It will be added for object properties in the near future.

### 4.8 Alternatives Vocabulary and Syntax

The main priority of the ongoing developments is to establish the principles of ADL as a language. Here, we refer to a language as a set of instructions to implement algorithms that produce various kinds of output through abstractions for defining and manipulating data structures or controlling the flow of execution. It is however important to distinguish that a language can be expressed using alternative vocabulary or syntax. Here, vocabulary is the words with a particular meaning in the language, such as block or keyword names, and syntax is the set of rules that defines the combinations of symbols that are considered to be a correctly structured expression of the language. Our experience on the way from CutLang v1 and LHADA to ADL showed that there might not always be a single best syntax for expressing a given content. Alternative syntax options may be more favorable in different use cases, due to practicality or simply due to different tastes of the users. Recognizing this, we recently opted to host multiple syntactic alternatives in ADL and CutLang for several cases. The most obvious case is the syntax for expression of object attributes, as described in Supplementary Appendix A.2. It should be noted that these alternatives can only exist for simple, localized syntactic expressions but not for the overall content and structure of the language. A more minor example is the name for the event classification block keyword, i.e. both region and algo are valid. Another is in the expression of specifying the input object collection in an object block, where either take keyword, using keyword or a colon “:” are valid. CutLang was recently updated to be able to parse and interpret different alternatives in such cases. We believe such flexibility will allow users to find the best ways to express their ideas and moreover will help CutLang to grow its overall user base.

## 5 Analysis Output

CutLang as an analysis framework is designed to output information and data that would be used for further analysis. The main output obtained after running an analysis in CutLang is provided in a ROOT file. The file, first of all, includes a copy of the ADL file content in order to document the provenance of the analysis. It also includes histograms with all the event counts and uncertainties obtained from the analysis and all histograms defined by the user. CutLang is also capable of skimming and saving events using the auxiliary save keyword in its internal format LVL0, as described in Supplementary Appendix A.10.3. In case event saving is specified in the ADL file, the ROOT file also stores the saved events.

The output ROOT file includes a directory for each event categorization region, i.e. each region block. These directories contain all user-defined histograms specified in the ADL file. The prototype version of CutLang also had a basic cutflow histogram listing the number of events surviving each step of the selection in the given region. The cutflows, including the statistical errors on counts are also given as text output. In the current version, the cutflow histograms are improved to include the selection criteria as bin labels. Moreover, in case binning is used in a region, a bincounts histogram is also added, where each histogram bin shows the event counts and errors in each selection bin, and the histogram bin labels show the bin definition. The cutflow and bincounts histograms can be directly used in the subsequent statistical analysis of the results. A screenshot of a simple example output can be seen in Figure 4 in Supplementary Appendix A.11.3.

### 5.1 Incorporation of Existing Counts

In some cases, event counts and uncertainties from external sources are needed to be systematically accessible in order to be processed together with the counts and uncertainties obtained from running the analysis via CutLang. One example is phenomenological interpretation studies, where the analysis is only run through signal samples, while the experimental results, consisting of data counts and background estimates are usually taken from the experimental publication. Having the data counts and background estimates directly available in a format compatible with the signal counts is necessary for subsequent statistical analysis. Moreover, for this particular case, it is also highly desirable to have this information documented directly within the ADL file. Another example is validation studies, when either multiple teams in an experimental group are synchronizing their cutflows, or a reimplemented analysis for a phenomenological interpretation study is validated against a cutflow provided by the original experimental publication. Similarly, having the validation counts and uncertainties in the same format would make comparison very practical.

Recently, a syntax was developed in ADL for systematically storing external counts and uncertainties within the ADL file. The physics process for which the information is given, and the format of the information is provided within the countsformat block using the process keyword, while the values are given in the relevant region blocks right after the definition of the relevant selection criteria using the counts keywords. The syntax is detailed in Supplementary Appendix A.11.2. When an ADL file including external counts and errors is run with CutLang, the counts and errors are converted into cutflow and bincounts histograms with a similar format to those hosting the CutLang output. The histogram and are placed under the relevant region directories, and physics process is included in the histogram names.

## 6 Performance and Multi-Threaded Runs

**FIGURE 2:**
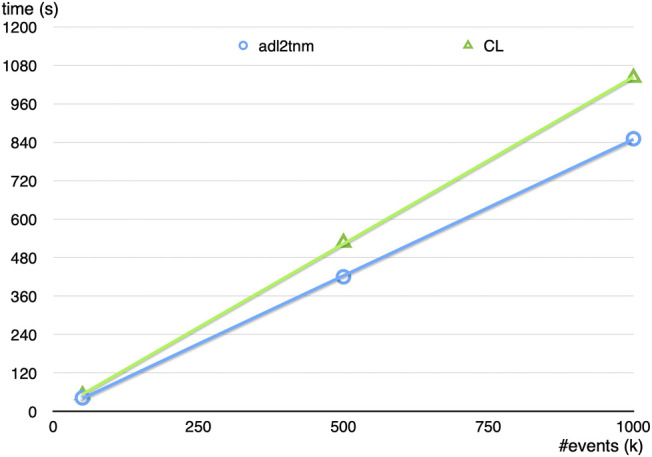
Speed comparison of CutLang versus the C++ ADL transpiler adl2tnm on a CMS supersymmetry analysis (CMS et al., 2019)^6^ using up to 1M supersymmetry signal events with the CMS NanoAOD format in a Mac OS setup.

The CutLang run-time interpreter is eventually aimed for use in the analysis of very large amounts of experimental data. Therefore its speed and performance needs to be close to those of analyses tools based on GPLs. It is expected that the process of run-time interpretation would decrease the performance due to additional tasks including lexical analysis, tokenization, etc. Yet, at its current state, CutLang’s speed is only partially less than that of a C++ analyzer. For a numerical test, a sufficiently complicated supersymmetry search analysis (CMS et al., 2019)^6^ involving multiple objects, 12 event categorization regions and several variable calculations based on external functions was run both with CutLang and the C++-based ADL transpiler adl2tnm using up to 1M supersymmetry signal events with the CMS NanoAOD format. The speed comparison for running in a Mac OS setup is shown in Figure 2. Overall, CutLang is about 20% slower compared to the same analysis performed using a pure C++ code.

CutLang has been also recently enhanced with the capability of multi-threaded execution of an analysis to optimally utilize the available resources and therefore get faster results. Adding -j n to the command to start the analysis execution enables using n number of cores, e.g., as

./CLA.sh [inputrootfile] [inputeventformat] -i [adlfilename] -j 2

for 2 cores. The requirement for n is to be an integer between 0 and the total number of cores on the processor, where the case of -j 0 is used to select one less than the total number of cores to maximize performance for demanding analyses while leaving the operating system necessary part of the resources.

**FIGURE 3:**
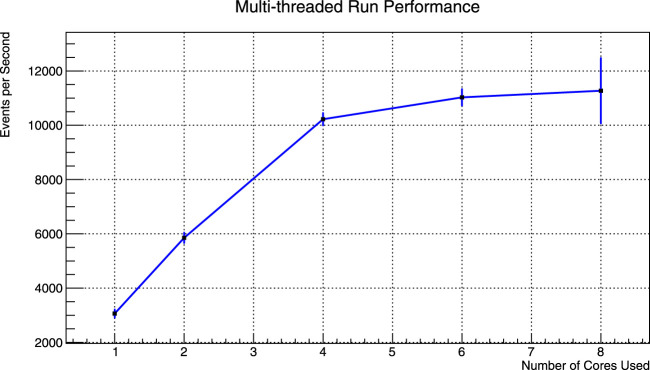
Events processed per second when analysis is divided into 1, 2, 4, 6 and 8 threads for varying number of events. Error bars are multiplied by 10 to make them visible.

Figure 3 shows the run time dependence on multi-threading. The mean and standard deviation of these results are further given in Table 2. The computer used during the test has Intel(R) Core(TM) i5-8300H with 4 cores, 8 threads and runs Ubuntu 18.04.4 LTS. The number of events analyzed was limited to 3 million due to memory restrictions in the current ROOT implementation. Although this is not the only possible way to collect results, it was convenient enough for a first implementation. It is surely possible to improve this implementation when the need arises by saving data on disk to free memory while continuing to run.

**TABLE 2:** Data points given in Figure 3.

Threads	Mean no. of events/s	SD
1	3,063.4	14.5
2	5,853.5	18.5
4	10,223.3	22.3
6	11,028.0	29.6
8	11,272.0	119.6

As can be seen from the results, total event processing rate increases linearly as the number of cores increase up to 4. Due to the processor having only 4 physical cores with 2 logical cores each, the runs that use more than 4 threads showed minimal improvement. Simultaneous processing efficiency, resource demand of background processes and recombination of results that are obtained in parallel also contribute to the decline in the multi-threaded run performance.

In a different performance test, run times for 1, 2, 4 and 8 threaded analyses for varying numbers of events are given in Table 3. To simplify, a normalized version of Table 3 is also provided in Table 4, where the run time of an analysis that used a single core is taken to be the norm. Looking at these tables, it can be seen that, as the analyses get more complex, higher levels of multi-threading performance gets increasingly better.

**TABLE 3:** Variation of run times with changing number of threads.

Processed events	Process time for core used (s)			
	1	2	4	8
$10^{4}$	3.081	3.041	3.124	4.600
$10^{5}$	21.085	12.062	8.316	9.630
$10^{6}$	306.064	155.195	91.201	97.968
$2.5\times 10^{6}$	776.133	402.723	227.817	209.623
$4.5\times 10^{6}$	1,409.416	722.901	416.964	374.946

**TABLE 4:** Runtimes as percentages of single core runtime.

Processed events	Normalized process time			
	1	2	4	8
$10^{4}$	100	98.7	101	149
$10^{5}$	100	57.2	38.6	45.7
$10^{6}$	100	50.7	29.8	32.0
$2.5\times 10^{6}$	100	51.9	29.4	27.0
$4.5\times 10^{6}$	100	51.3	29.6	26.6

A simple analysis task uses time mainly on reading data from disk and performing memory transfers. One should note that having a multicore system does not make an extra contribution in this scenario as there is only one disk. If the analysis becomes more complicated, the impact of read and copy operations gets reduced and CPU-intensive calculations start taking more time. Therefore in a CPU-intensive complex analysis, the benefit of having multiple cores becomes more pronounced.

## 7 Code Maintenance and Continuous Integration

The CutLang source code is public and resides in the popular software development platform GitHub^7^:

https://github.com/unelg/CutLang

CutLang uses GitHub functionalities for parallel code development across multiple developers. This development platform, apart from a wiki page for documentation and possibility for error reporting, also offers a continuous integration setup which includes a series of tasks that could be initiated at a specific time or by a trigger such as a commit to the main branch. The continuous integration setup was recently incorporated to automatically validate the code. The setup compiles the CutLang source code from scratch, and runs the resulting executable over a set of example ADL files from the package on a multitude of input data files and formats. By comparing the output from the examples to a reference output from earlier runs that were successfully executed and validated, any coding errors could be automatically detected and reported by email. The total compilation and execution time is greatly reduced by using a pre-compiled version of ROOT and by pre-installing the necessary event files onto a Docker^8^ image integrated to a recent Linux (Ubuntu) virtual computer made available by the development platform.

## 8 Analysis Examples

ADL and CutLang are continuously being used for implementing a diverse set of LHC analyses and running these on events. The analyses implemented are being collected in the following GitHub repository^9^:

https://github.com/ADL4HEP/ADLLHCanalyses

The main focus so far has been to implement analyses designed for new physics searches, in particular supersymmetry searches. These supersymmetry analyses are intended to be directly used to create model efficiency maps to be used by the reinterpretation framework SModelS (Kraml et al., 2014; Ambrogi et al., 2018; Ambrogi et al., 2020). The results obtained by running some of the implemented analyses have also been validated within dedicated exercises performed during the Les Houches PhysTeV workshops, in comparison to other analysis frameworks (Brooijmans et al., 2020). The available analysis spectrum is currently being extended to cover Higgs and other SM analyses. Furthermore, studies are ongoing to improve the functionalities of ADL and CutLang for use in searches or interpretation studies with long-lived particles, which involve highly non-conventional objects and signatures. More recently, analyses examples for CMS Open Data^4^ and a sensitivity study case for High Luminosity LHC and the Future Circular Collider were also added (Paul et al., 2021). In addition, ADL and CutLang were used as main tools in an analysis school which took place in Istanbul in February 2020 for undergraduate students, and several analyses were implemented by the participating students (Adiguzel et al., 2008). ADL and CutLang were also used to prepare hands-on exercises for data analysis at the 26th Vietnam School of Physics (VSOP) in December 2020.^10^ The VSOP exercises involving running CutLang and further analysis of resulting histograms with ROOT were also adapted for direct use via Jupyter notebooks, and are documented in detail in VSOP hands-on exercises.^11^ The experience in both schools justified ADL and CutLang as highly intuitive tools for introducing high energy physics data analysis to undergraduate and masters students with nearly no experience in analysis.

Implementing analyses with a variety of physics content led to incorporating a wider range of object and selection operations and helped to make the ADL syntax more generic and inclusive. Syntax for generalizing object combinations, numerical efficiency applications, hit-and-miss method, bins and counts and many others were introduced as a result of these studies. Consequently, the scope and functionality of CutLang interpreter and framework was also enhanced. Many internal and external functions were added to CutLang to address direct requirements of the various implemented analyses. Running different analyses on events also allowed to thoroughly test the capacity of CutLang in performing complete, realistic analysis tasks.

## 9 Conclusions

We presented the recent developments in CutLang, leading towards a more complete analysis description language and a more robust runtime interpreter. The original syntax of the earlier CutLang prototype version and its event processing methods have been modified after a multitude of discussions with other scientists in the field interested in decoupling the physics analysis algorithms from the computational details and after implementing many HEP analyses. Modifications include significant enhancement of object definition and event classification expressions, addition of more functions for calculating event variables, incorporation of tables for applying efficiencies, adaptation of a system for including external counts, and more. Although these modifications broke the strict backward compatibility of the earlier version of the language, we believe they should be considered as improvements as they certainly will lead to a cleaner, more robust and a widely accepted analysis description language. The improved syntax processing relies on formal lexical and grammar definition tools widely available in all Unix-like operating systems.

One direct result of the syntax modifications originating from community-wide discussions is that, in the presented version there are more than a single way of expressing the same idea in CutLang. We believe this is a desirable property: after all, in human languages (that we try to imitate) as well, the same idea can be expressed in multiple ways. To give an example to reject events with a property smaller than a certain threshold amounts to accepting events greater than the same threshold. Such a property should not be considered as a source of potential confusion and error, but as a fertility of the language.

CutLang still follows the approach of runtime interpretation. We strongly believe that direct interpretation of the human readable commands and algorithms, although slower in execution as compared to a compiled binary, leads to faster and less error-prone algorithm development. The possible event processing speed issues can be cured by parallel processing of independent events and regions. The interpreted and human readable nature of CutLang and ADL have a potential area of growth and development: with the advance of machine learning hardware and software tools, the dream of being able to perform an LHC-type analysis just by talking to the computer in one’s native tongue might not be too far-fetched.

The advances described in this paper brought ADL and CutLang to a state where they can handle many standard analysis expressions and operations and have developed the earlier prototype into a practically usable infrastructure. CutLang at its current stage can directly perform phenomenological studies and some simple experimental studies. However there are still some limitations to address in the language and the interpreter. In the near future, ADL syntax will be further expanded by inclusion of a generic way to describe arbitrary combinations of objects to form new ones, the capability of adding new object attributes and defining object associations, lower level objects or non-standard objects such as long-lived particles. One major addition would be the capability to express and handle variations due to systematic uncertainties. Moreover, the interpreter would benefit from further automatizing the incorporation of new input data types or external functions, which currently require manual intervention from the users. Enabling an automated syntax verification and providing explicit guidance for possible syntax errors would further facilitate the analysis process. Plans are underway to improve the design of the CutLang infrastructure in the near future based on current best practices in compiler construction to accommodate all these features and arrive at a more robust, yet flexible and user-friendly analysis ecosystem. With the growing data, our field will undoubtedly continue conceiving new analysis concepts and methods which may not be immediately applicable in ADL and CutLang. The current developer team is dedicated to following and implementing these features. Yet, we foresee that the planned improvements in the fundamental design of ADL and CutLang will lead the progress towards the ultimate goal of analysis automation.

Finally, as any language, CutLang/ADL grows with the people that use it to solve new problems. With every analysis requiring a new functionality, the list of already-solved problems grows. We hope that, such an internal library together with the script assisted addition of external user functions will allow the analysts of the future to spend less time on previously solved problems and to focus their energy in innovating solutions to the analysis problems of the post LHC era colliders.

## Data Availability

The original contributions presented in the study are included in the article/Supplementary Material, further inquiries can be directed to the corresponding author.

## Footnotes

1 “SciPy: Scientific Computing Tools for Python.”

2 “Workshop: Analysis Description Languages for the LHC, 6–8 May 2019, Fermilab LHC Physics Center.” https://indico.cern.ch/event/769263/.

3 “Lex and Yacc Page.” http://dinosaur.compilertools.net.

4 “CERN Open Data Portal.” http://opendata.cern.ch.

5 This block was called algo in the original CutLang syntax. Even though algo is still valid in CutLang, we generally refer to the block as region, as the latter is a more domain specific word.

6 “ADL Implementation of the CMS SUSY Razor Analysis.” https://github.com/ADL4HEP/ADLLHCanalyses/tree/master/CMS-SUS-16-017.

7 “CutLang GitHub Repository.” https://github.com/unelg/CutLang.

8 “Docker Web Page.” https://www.docker.com/.

9 “ADL LHC Analyses Repository.” https://github.com/ADL4HEP/ADLLHCanalyses.

10 “26th Vietnam School of Physics: Particles and Dark Matter, 29 Nov 2020–11 Dec 2020, Quy Nhon.” https://indico.in2p3.fr/event/19437/overviewhttps://indico.in2p3.fr/event/19437/overview.

11 “VSOP Hands-On Exercises.” https://github.com/unelg/CutLang/wiki/VSOP26HandsOnEx.
