# Supplementary material for: CutLang v2: Advances in a Runtime-Interpreted Analysis Description Language for HEP Data
Source: Front Big Data. 2021 Jun 7;4:659986. doi: 10.3389/fdata.2021.659986 (PMC8218547; doi:10.3389/fdata.2021.659986)
Supplement: Supplementary file 1 [file DataSheet1.pdf]

Appendices: CutLang v2: Advances in a runtime-interpreted analysis description language for HEP data

G. Unel[1], S. Sekmen[2], A. M. Toon[3], B. Gokturk[4], B. Orgen[4], A. Paul[5], N. Ravel[6], and J. Setpal[7]

[1]University of California at Irvine, Department of Physics and Astronomy, Irvine, USA
[2]Kyungpook National University, Department of Physics, Daegu, South Korea
[3]Saint Joseph University of Beirut, Dept. of Computer Software Engineering, Beirut, Lebanon
[4]Bogazici University, Department of Physics, Istanbul, Turkey
[5]The Abdus Salam International Centre for Theoretical Physics, Trieste, Italy
[6]University of Ankatso, Department of Physics, Antananarivo, Madagascar
[7]R.N. Podar School, Mumbai, India

May 31, 2021

Contents

A User Manual **2**
A.1 Blocks and keywords . . . 2
A.2 Predefined physics objects . . . 2
A.3 Predefined functions . . . 3
A.3.1 PDGID of particles . . . 5
A.4 Mathematical operators and functions . . . 5
A.5 Comparison, range and logical operators . . . 6
A.5.1 Logical operations . . . 6
A.5.2 Ternary operator . . . 6
A.6 χ^2 minimization . . . 6
A.7 Definitions . . . 7
A.8 Tables . . . 7
A.9 Manipulating objects . . . 8
A.9.1 Defining new objects . . . 8
A.9.2 Sorting objects . . . 8
A.9.3 Object combinatorics . . . 8
A.9.4 Looping over a subset of the object collection . . . 9
A.9.5 Minimum and maximum of object attributes . . . 10
A.9.6 Summing object attributes . . . 10
A.9.7 Object constituents . . . 10
A.9.8 Daughter particles . . . 10
A.9.9 Hit and miss method . . . 10
A.10 Manipulating Events . . . 11
A.10.1 Selecting or rejecting events . . . 11
A.10.2 Weighing events . . . 11
A.10.3 Saving events . . . 11
A.11 Bins, counts and histograms . . . 11
A.11.1 Bins . . . 11
A.11.2 Counts . . . 12
A.11.3 Histograms . . . 12
A.12 Structure of a complete ADL file . . . 13

A.12.1 Initialization and information section 13
A.12.2 Regions and algorithms 13

B The CutLang framework **14**
B.1 installation and compilation 14
B.2 External user functions 14
B.3 Incorporation of new input file types 15

A User Manual

All information about ADL and CutLang including publications, talks and twikis with syntax rules can be accessed through the following portal

https://cern.ch/adl

The code for CutLang is hosted in the GitHub repository

https://github.com/unelg/CutLang

which provides up-to-date instructions on how to install, compile and run CutLang .

A.1 Blocks and keywords

An ADL file consists of blocks based on a keyword value/expression structure. The blocks allow a clear separation of analysis components. A typical block looks as follows:

```
blockkeyword blockname
    # general comment
    keyword1 expression1
    keyword2 expression1
    keyword3 expression1 # comment about value3
```

Table 2 lists the available blocks, their purposes and associated keywords, and Table 1 lists the keywords. The details on their applications are given in the following sections.

Table 1: Blocks in ADL and CutLang

Block	Purpose	Related keywords
object / obj	Object definition block. Produces an object type from an input object type by applying selections.	take, select, reject
region / algo	Event categorization.	select, reject, weight, bin, sort, counts, histo, save
info	Contains analysis information such as the experiment, center-of-mass energy, luminosity, publication details, etc.	
table	Generic block for tabular information, such as efficiency values versus variable ranges	tabletype, nvars, errors
countformat	Expresses the processes for which external counts are included and the format of counts	process

A.2 Predefined physics objects

Basic physics objects and their properties currently available in CutLang are defined in Table 3. The predefined particles are initially sorted per decreasing transverse momentum and their indices start at zero. With the current implementation, all the predefined particle names, and commonly used function names have become case-insensitive. For the particle, both Python-type and LaTeX-type notations are accepted; the former with square brackets, and the latter with an underline character. An example for electrons is given below:

Table 2: Keywords in ADL and CutLang

Keyword	Purpose	Related block
define	Define variables, constants	–
select	Select objects or events based on criteria that follow the keyword.	object, region
reject	Reject objects or events based on criteria that follow the keyword.	object, region
take / using / :	Define the mother object type	object
sort	Sort an object in an ascending or descending order wrt a property.	region
weight	Weight events	region
histo	Fill histograms	region
process	Specify process and the format for which external counts are given	countformat
counts	Give external counts	region
tabletype	Specifies type of the table	table
nvars	Number of variables in a table	table
errors	Type of errors indicated in a table	table
title, experiment, id, publication, sqrtS, lumi, arXiv, hepdata, doi	Provide information about the analysis (see Table 12)	info

`Ele_0  = ELE_0 = Ele[0] = ele[0] = electron_0 = electron[0]` .

Sometimes it is necessary to refer to the whole object set or just to some of its members. The CutLang notation for these cases is to write the name of the set without any indices for the former (i.e. `ELE`) and to use the semi-colon notation for the latter (i.e. `ELE[0:2] = ELE_0:2`) .

In CutLang , there are two object-types that merit special attention: the lepton and the neutrino types. The `LEP` keyword refers to a generic lepton and at runtime it is reduced to an electron or to a muon depending on the choice as explained in Table **??**. This helps the physicist avoiding two algorithm sections, one for electron and other muon based analyses. The second object-type is related to the taming of the neutrino escaping from the detector. At LHC energies and beyond, for which CutLang is intended, the W bosons are generally produced with a sufficient boost such that in the leptonic decays, the pseudorapidity of the charged lepton is not very different from the chargeless one. Therefore this particular physics object benefits from this approximation to define a massless and chargeless particle with transverse momentum and azimuthal angle (ϕ) values extracted from the missing transverse energy (MET) measurements. The pseudorapidity, however, is taken equal to that of the charged lepton with the same particle index.

Table 3: Basic physics object nomenclature in CutLang

Name	Keyword	First object		Second object		$j+1^{th}$ object
Electron	ELE	ELE[0]	ELE_0	ELE[1]	ELE_1	ELE_j
Muon	MUO	MUO[0]	MUO_0	MUO[1]	MUO_1	MUO_j
Tau	TAU	TAU[0]	TAU_0	TAU[1]	TAU_1	TAU_j
Lepton	LEP	LEP[0]	LEP_0	LEP[1]	LEP_1	LEP_j
Photon	PHO	PHO[0]	PHO_0	PHO[1]	PHO_1	PHO_j
Jet	JET	JET[0]	JET_0	JET[1]	JET_1	JET_j
Fat Jet	FJET	FJET[0]	FJET_0	FJET[1]	FJET_1	FJET_j
b-tagged Jet	BJET	BJET[0]	BJET_0	BJET[1]	BJET_1	BJET_j
light Jet	QGJET	QGJET[0]	QGJET_0	QGJET[1]	QGJET_1	QGJET_j
Neutrino	NUMET	NUMET[0]	NUMET_0	NUMET[1]	NUMET_1	NUMET_j
MET	METLV	METLV[0]	METLV_0	—	—	—
generator particle	GEN	GEN[0]	GEN_0	GEN[1]	GEN_1	GEN_j

A.3 Predefined functions

Functions in CutLang can be used for accessing object attributes, or for computing new variables from object or event quantities. Functions for accessing object attributes can be directly related to Lorentz vectors such as mass, momentum, rapidity etc, or be related to other variables found in some commonly used ntuples. In

both cases, both the function syntax with parentheses and the attribute syntax with curly braces can be used. Functions used for computing new quantities can use object attributes or other already calculated quantities or constants. The currently available object attribute functions in CutLang are listed in Table 4. Note that some of the attributes listed here are only valid for certain input types, e.g. for CMS NanoAOD, but not for others, e.g. for Delphes. The functions used for computing new quantities are listed in Table 5.

One should note that in CutLang adding particles could be achieved by either writing these one after the other separated by space(s), or by using a + sign. Both notations are equally valid. Additionally, one should use a comma as the separation character for the functions requiring multiple arguments.

The internal functions, such as angular distance or transverse momentum are also case-insensitive in CutLang , though they are written in this manuscript with a certain syntax (first letter upper case) for clarity in reading. The functions requiring multiple arguments should use comma character for argument separation. External functions can also be downloaded and added to CutLang library. The instructions for this operation is described in appendix B.

Table 4: Functions and syntax for object attributes in CutLang .

**Meaning**	Syntax 1	Syntax 2
Lorentz vector-related attributes		
Mass of	`m( )`	`{ }m`
Charge of	`q( )`	`{ }q`
Phi of	`Phi( )`	`{ }Phi`
Eta of	`Eta( )`	`{ }Eta`
Absolute value of Eta of	`AbsEta( )`	`{ }AbsEta`
Rapidity of	`Rep( )`	`{ }Rep`
Pt of	`Pt( )`	`{ }Pt`
Pz of	`Pz( )`	`{ }Pz`
Energy of	`E( )`	`{ }E`
Momentum of	`P( )`	`{ }P`
Other attributes		
PDGID of a particle	`PDGID( )`	`{ }PDGID`
Charge of a particle	`btagDeepB( )`	`{ }btagDeepB`
is the jet b tagged?	`bTag( )`	`{ }bTag`
Soft Drop mass of a jet	`msoftdrop( )`	`{ }msoftdrop`
N-subjetiness variable 1	`tau1( )`	`{ }tau1`
N-subjetiness variable 2	`tau2( )`	`{ }tau2`
N-subjetiness variable 3	`tau3( )`	`{ }tau3`
Leptonic diTau invariant mass	`fMTauTau( )`	`{ }fMTauTau`
transverse impact parameter	`dxy( )`	`{ }dxy`
longitudinal impact parameter	`dz( )`	`{ }dz`
lepton identification variable	`softId( )`	`{ }softId`
relative isolation for leptons	`miniPFRelIsoAll( )`	`{ }miniPFRelIsoAll`
MVA based tau ID	`dMVAnewDM2017v2( )`	`{ }dMVAnewDM2017v2`
$\sigma_{i\eta i\eta}$ for photons	`sieie( )`	`{ }sieie`
isolation variable	`reliso( )`	`{ }reliso`
isolation variable	`relisoall( )`	`{ }relisoall`
isolation variable	`pfreliso03all( )`	`{ }pfreliso03all`
Tau decay mode id	`iddecaymode( )`	`{ }iddecaymode`
Tight ID and isolation flag	`idisotight( )`	`{ }idisotight`
Tight anti ele ID for taus	`idantieletight( )`	`{ }idantieletight`
Tight anti mu ID for taus	`idantimutight( )`	`{ }idantimutight`
Tight ID for muons	`tightid( )`	`{ }tightid`
PU ID for jets	`puid( )`	`{ }puid`
Index of matched genparticle to a lepton	`genpartidx( )`	`{ }genpartidx`
Tau decay mode	`decaymode( )`	`{ }decaymode`
Tau isolation	`tauiso( )`	`{ }tauiso`
Muon soft ID	`softId( )`	`{ }softId`

Table 5: Functions and syntax for computing new quantities in CutLang .

**Meaning**	Syntax 1	Syntax 2
Angular distance between	`dR( )`	{ }`dR`
Phi difference between	`dPhi( )`	{ }`dPhi`
Eta difference between	`dEta( )`	{ }`dEta`
Missing transverse energy in the event	`MET`	–
sum of jet transverse momenta	`HT( )`	–
partitioning objects into 2 megajets	`fmegajets( )`	{ }`fmegajets`
Razor variable MR	`fMR( )`	{ }`fMR`
Razor variable MTR	`fMTR( )`	{ }`fMTR`
partitioning objects into 2 hemispheres	`fhemisphere( )`	{ }`fhemisphere`
transverse mass MT2	`fMT2( )`	{ }`fMT2`

A.3.1 PDGID of particles

Each type of particle recognized in particle physics is assigned a unique code by the Particle Data Group (PDG) in order to facilitate interface between event generators, detector simulators, and analysis packages. These codes are known as PDGID (or PDG ID), and this method is called the MC particle numbering scheme [1]. The numbering includes elementary particles such as, electrons, neutrinos, Z bosons etc, composite particles (mesons, baryons etc) and atomic nuclei. Hypothetical particles beyond the Standard Model also have PDGIDs. Particles have a positive PDGID whereas antiparticles a negative one. The list of PDGID of some particles is given in table 6

Table 6: PDGID of some elementary particles[2]

Quarks		Leptons		Bosons	
d	1	e^-	11	γ	22
u	2	μ^-	13	Z	23
s	3	τ^-	15	W^+	24

CutLang provides an internal function that obtains a particle's PDGID. Particles of a certain type can be selected using this functionality, e.g. :

```
select  PDGID( LEP[0]) == -11
```

This command selects positrons. (Positron is the antiparticle of electron, therefore it has a negative PDGID)

A.4 Mathematical operators and functions

Mathematical functions available in CutLang are listed in Table 7. Trigonometric and logarithmic functions are implemented with their usual meanings. The Heaviside step function or the unit step function `hstep`, which was also added recently, is a discontinuous function, named after Oliver Heaviside, whose value is zero for negative arguments and one for positive arguments. The reducer functions for minimization and maximization, `min` and `max`, which were added recently, are discussed in Appendix A.9.5. The reducer function `size / count` returns the number of elements of a given set, such as the number of electrons.

Table 7: mathematical and logical operators

Meaning	Operator	Meaning	Operator
number of	Size( ) Count() NumOf()	absolute value	abs()
tangent	tan()	hyperbolic tangent	tanh()
sine	sin()	hyperbolic sine	cosh()
cosine	cos()	hyperbolic cosine	sinh()
natural exponential	exp()	natural logarithm	log()
square root	sqrt()	Heaviside step function	hstep()
as close as possible	˜=	usual meaning	+ - / *
as far away as possible	˜!	to the power	ˆ

A.5 Comparison, range and logical operators

CutLang understands the basic mathematical comparison expressions and logical operations. `C/C++` operator notations and their Fortran counterparts are recognized and correctly interpreted. Additionally square brackets are used to define inclusive or exclusive ranges. The available comparison, range and logical operators can be found in Table 8.

Table 8: Comparison, range and logical operators in CutLang

Keywords	Explanation
`> >= == <= <`	usual meaning
`GT GE EQ LE LT`	usual meaning
`!= NE`	not equal
`[ ]`	in the interval
`] [`	not in the interval
`NOT`	logical not
`AND and &&`	logical and
`OR or ||`	logical or

A.5.1 Logical operations

The use of Boolean operators (AND, OR, NOT) can make it easy to write the event selection criteria. In CutLang , logical AND and logical OR operator had already been used to combine multiple event selection criteria. The newly implemented logical NOT simplifies the way to write the criteria of event selections in the analysis code to a great extent. The simplest example code to understand the syntax:

```
select NOT Size(ELE) > 4
```

This command selects events which do NOT have number of electrons greater than 4. However, the advantage of the NOT operator becomes more apparent when trying to negate more complex selections. The event selection criteria can be combined using the logical AND, OR, NOT. For example :

```
select (NOT condition1 ) AND ( condition2 OR condition3 )
```

Now let us look at another code :

```
select Size(ELE) == 2
select NOT ( {ELE[0] ELE[1]}q == 0 AND {ELE[0] ELE[1]}m [] 80 100)
```

The criteria `( {ELE[0] ELE[1]}q == 0 AND {ELE[0] ELE[1]}m [] 80 100)` can be used for defining Z bosons. As we have set NOT, we veto events with Z boson while looking for other dilepton signatures. Without using the `NOT` command, this selection would not be so straightforward, and would require a more complicated expression.

A.5.2 Ternary operator

Application of conditional selection criteria is available, including nested statements, using a syntax similar to that of C++ :

```
condition ? true-case : false-case
```

The following example illustrates a use case: if the number of `muonsVeto` particles equals to 1, then the `MTm` quantity should be less than 100 otherwise the `MTe` quantity should be less than 100:

```
Size(muonsVeto) == 1 ? MTm < 100 : MTe < 100
```

A.6 χ^2 minimization

In an analysis with a multitude of objects of the same type, the analyst could search for the best combination defined by some criterion. A typical example, used in fully hadronic $t\bar{t}$ reconstruction would be to find the jet combination that would yield the best W boson mass, or to find the two charged leptons that would result in the best Z boson mass. The need for such a search can be expressed in CutLang using two special

comparison operators: `~=` and `~!`. The former is used in the sense of "as close as possible to" whereas the latter for calculation "as far as possible from". These two operators can be used to express χ^2 minimization kinds of operations. The indices of the particles in such a search are to be given as negative. For example, the statement "find two leptons with a combined invariant mass as close to 90.1 GeV" can be expressed in CutLang notation as { `LEP_-1 LEP_-1` }`m ~= 90.1` . In this case, CutLang finds the best pair of particles satisfying the condition, and stores it per event for possible later use. However the analyzer should not use negative indices directly inside the `region` block. It is a much better practice that improves readability to define a new object such as `define ZLepRec = LEP[-1] LEP[-1]`. This definition can be used when defining histograms or other selection criteria, such as when selecting the charge of the found lepton pair, etc. If another particle of the same type (e.g. another lepton) is to be found, it is necessary to use a different but still negative index value.

A.7 Definitions

ADL and CutLang allow to assign alias names to constants (e.g. Z boson mass) or variables (e.g. angular variables between objects, mass of the Z boson reconstructed from two leptons, etc.). The syntax and examples are given in Table 9. Note that the keyword `define` can also be shortened as `def`.

Table 9: Simple definitions

Keywords	argument1	symbol[1]	argument1	Example
`define`	name	:/=	value	define mZprime = 500
`define`	name	:/=	function	define mTop1 : m(Top1)
`define`	name	:/=	particle(s)	define Zreco : ELE[0] ELE[1]

A.8 Tables

The present version of CutLang incorporates tables to implement various HEP related quantities, such as efficiencies, acceptances or trigger turn-on curves. Currently only one and two-dimensional tables can be used. These tables should have a name and a table type, specified by the `tabletype` keyword, where the latter defines what information is hosted by the table. Currently, only efficiency tables are recognized, therefore the table type information only serves as documentation and is not used by the interpreter. However, as other uses for tables are developed, table type would become more relevant in the future. Tables must also specify the number of variables (1 or 2) using the `nvars` keyword as well as the availability of errors on the central value (true or false) using the `errors` keyword. These should be followed by the table data, using the value [lower-error upper-error] lower-limit1 upper-limit1 [lower-limit2 upper-limit2] notation. Once defined in the definitions section, the table can be referred to and used in object and event selection. An example table is shown below:

```
table tightmuoneff
      tabletype efficiency
      nvars 2
      errors true
#     value  err-  err+     min    max       min    max
        0.1  0.01  0.02      0.0   10.0      -5.5    0.0
        0.1  0.01  0.02      0.0   10.0       0.0    5.5
        0.2  0.01  0.03     10.0   20.0      -5.5    0.0
        0.2  0.01  0.03     10.0   20.0       0.0    5.5
        0.4  0.01  0.04     20.0   50.0      -5.5    0.0
        0.4  0.01  0.04     20.0   50.0       0.0    5.5
        0.7  0.01  0.05     50.0   70.0      -5.5    0.0
        0.7  0.01  0.05     50.0   70.0       0.0    5.5
        0.95 0.01  0.06     70.0   1000.0    -5.5    0.0
        0.95 0.01  0.06     70.0   1000.0     0.0    5.5
```

[1]both : and = can be used interchangeably

A.9 Manipulating objects

A.9.1 Defining new objects

New objects can be declared using a simple syntax:

```
object new_object_name : base_object_name
```

where the `object` keyword can also be shortened as `obj`, and instead of the symbol `:`, the keywords `using` and `take` can be used. The base object name can be a base object class, or a previously defined new object type such in the case of defining b-tagged jets from already defined high transverse momentum jets. These are usually called derived objects. An example, defining a derived new electron type based on predefined electrons would be written as:

```
obj goodEle : ELE
```

One way of defining a derived object type is to list a set of selection critieria that distinguishes it from the base object, such as:

```
object AK4jets
    take JET
    select {JET_}Pt > 30
    select {JET_}AbsEta < 2.4
```

It is also possible to create a new object by forming a group out of multiple base or derived objects, for example, to create a lepton object from electrons and muons. This is achieved using the `Union` function, as shown below. This particular case of new object creation does not use any selection.

```
object leps : Union( MUO , ELE, TAU) # add all leptons into a set

object gleps : Union( goodEle , goodMuo ) # add all derived leptons into another set
```

A.9.2 Sorting objects

By default, objects are sorted according to their transverse momentum, p_t, in descending order. For example, `ELE[0]` denotes the electron having the highest transverse momentum. In some cases, objects may need to be sorted according to some other property, such as energy, pseudorapidity etc. In the current version, this can be done as:

```
    sort {ELE_ }E ascend
```

This command sorts electrons according to their energy in ascending order, i.e. `ELE[0]` will have the least energy. Sorting can also be done in the descending order by using `descend`.

A.9.3 Object combinatorics

Let us assume that we have an event with 5 jets, and we would like to reconstruct all hadronic Z bosons in the event. What are the combinations? Numbering the jets from 1 to 5, some possibilities are given in Table 10, in the left panel. It is obvious that not all possibilities are listed, and finally only one possibility can be true: after all a jet can not be used to reconstruct two different Z bosons. On top of this, other requirements might be applied to further restrict the possible Z candidates. For example, there might be a pseudorapidity range limit on each candidate, the transverse momentum of the jets forming the Z boson could be limited, the angular separation between the hadronic Z candidate and the first constituent jet might be limited, and finally, the invariant mass of the Z candidate might be requested to be in a certain range. After all these restrictions, the same initial set might be reduced to the combinations listed in the right panel of Table 10, where the candidates that did not pass the requirements are shown as stroked out.

Table 10: Combining two jets to reconstruct a hadronic Z boson

possibility ID	Z_1	Z_2
1	j_1j_2	j_3j_4
2	j_1j_2	j_3j_5
3	j_1j_2	j_4j_5
4	j_1j_3	j_2j_4
5	j_1j_3	j_2j_5
6	j_1j_3	j_4j_5
...	...	...

possibility ID	Z_1	Z_2
1	j_1j_2	~~j_3j_4~~
2	j_1j_2	j_3j_5
3	j_1j_2	~~j_4j_5~~
4	j_1j_3	j_2j_4
5	j_1j_3	j_2j_5
6	j_1j_3	j_4j_5
...	...	...

This combination example can be written in CutLang as:

```
object hZs : COMB( jets[-1] jets[-2] ) alias ahz #
the candidate is temporarily called ahz}
    select {ahz}AbsEta < 3.0
    select {jets[-2]}Pt > 2.1
    select {jets[-1]}Pt > 5.1
    select {jets[-1], ahz }dR < 0.6 # dR between ahz and its constituent 1, apply to all
    select {ahz}m [] 10 200
```

In order to activate this new object, and eliminate the combinations that do not satisfy the requirements, one has to put a selection command into the running algorithm (or region); this could be, for example, to have at least two hadronic Z candidates per event:

```
algo testCombinations
    select Size(jets) >= 2 #we need at least 2 jets for a Z boson
    select Count(hZs) >= 2 #the object name is used, not the temporary alias.
```

As indicated by Table 10 right side, there are still multiple possibilities, such as rows 2, 4 and 5. To further reduce these by killing the overlapping candidates and leave a single valid one, some sort of ideal condition should be specified. This can be achieved using the previously discussed χ^2 minimization. As an example case, let us require the masses of both candidates to be as close as possible to the known Z mass. Now, the final algorithm is given as:

```
object hZs : COMB( jets[-1] jets[-2] ) alias ahz #
the candidate is temporarily called ahz
    select { ahz }AbsEta < 3.0
    select {jets[-2] }Pt > 2.1
    select {jets[-1] }Pt > 5.1
    select {jets[-1], ahz }dR < 0.6 # dR between  ahz and its constituent 1, apply to all
    select { ahz }m [] 10 200

define zham : {hZs[-1]}m
define zhbm : {hZs[-2]}m
define chi2 : (zham - 91.2)^2 + (zhbm - 91.2)^ 2}

region testCombinations
    select ALL # to count all events # count number size are all the same.
    select Size(jets) >= 2 # we need at least 2 jets for a Z boson
    select Count(hZs) >= 2 # we need at least 2 Zhad candidates.
    select chi2 ~= 0 \# we kill here overlapping candidates. .
```

A.9.4 Looping over a subset of the object collection

By default, CutLang loops over all objects in a given collection. However, sometimes it is necessary to loop only over a subset, such as looping only through the first 3 jets. ADL and CutLang allow to specify the subset, e.g. as `jets[0:3]`.

A.9.5 Minimum and maximum of object attributes

Looping over objects can be used for selecting the minimum or maximum of a function based on any object attribute. An explanatory example could be to apply a selection based on the minimum value of the angular separation between each of the three most energetic jets and the most energetic electron. In CutLang , this criterion can be expressed as:

```
select  Min( dR(JET[0:2], ELE[0] )) > 0.9  .
```

A.9.6 Summing object attributes

CutLang allows looping over an attribute to calculate the sum of their values. A typical example would be the sum of transverse momenta of a set of jets. Although this frequently used function is predefined and available as HT, it could also be written as:

```
select  Sum( pT(JET) ) >= 20   .
```

A.9.7 Object constituents

Sometimes, the analysis might necessitate a selection based on jet constituents. CutLang allows the modifier word `constituents` only in case of jets (or any other jet-like objects, such as the large radius FatJets) to refer to these. An example for defining a new jet object based on criteria on the constituents would be:

```
object goodJet using JET
    select q(JET constituents ) == 0  #select neutral constituents
    select Sum(pT(JET constituents ) ) < 40  # PT from remaining constituents
```

Here the first criterion removes all the charged constituents of each jet and eventually the jet itself if it has no more constituents left, whereas the second criterion imposes an upper limit of 40 GeV to the sum of the transverse momenta of the remaining constituents of each jet. All other functions available in CutLang would work in the same way.

A.9.8 Daughter particles

While defining a new particle based on MC truth information, it is sometimes necessary to access the daughters of a given particle. CutLang is capable of accessing the daughters of of an MC truth particle. In the following example, the first selection criterion filters the particles that decayed into two or more daughters, while the second criterion is used to select only the daughters with electric charge.

```
object DVcandidates take GEN
    select daughters( GEN ) > 1     # 1 child not accepted
    select abs(q(GEN daughters) ) > 0  # charged daugters only
```

A.9.9 Hit and miss method

The `ApplyHM` function can be used to define new objects which pass or fail the efficiency test in that particular region of the parameter space. In CutLang , the random number generation is achieved via the TRandom3 [3] function in ROOT libraries. This function reports the time cost of the call to be about 5 ns on an Intel i7 CPU running at 2.6 GHz.

An example for electrons recorded by an imaginary detector whose electron detection efficiency is described by a table called `myDet` can be written as:

```
object myElectron
    take ELE
    select applyHM( myDet({ELE}pT , {ELE}Eta) == 1) # 0 to reject, 1 to accept.
```

The analysis algorithm can make use of this newly defined object, `myElectron`, to apply selection criteria, such as the available number of electrons per event etc.

A.10 Manipulating Events

A.10.1 Selecting or rejecting events

The conditions based on which an event can be selected or rejected are written in the `region / algo` blocks. They start with the `select` or `reject` keywords, and are expressed in the form of functions applied on particles complemented by a comparison operator and a limit value. An example for `select` would be

```
select Size(goodEle) >= 2}
```

The synonyms `cut` and `cmd` can be interchangeably used in place of the `select` keyword. The keyword `reject` is equivalent to `select not`, thus rejecting the events that match the given criteria, as in the example below:

```
reject  {ELE[0] ELE[1]}q == 0 AND {ELE[0] ELE[1]}m [] 80 100
```

There are also some special keywords that require further discussion. These are shown in Table 11. Select `ALL` accepts all events, for example it can be used for event counting purposes. The next two are scale factors mostly used in ATLAS related analyses. For other input file formats these scale factors are automatically set to unity.

Table 11: Special Conditions in CutLang

Keywords	Example	Explanation
`ALL`	`select ALL`	accept all events
`LEPsf`	`cmd LEPsf`	apply leptonic scale factor to MC events
`bTagSF`	`cmd bTagSF`	apply b-jet tagging scale factor to MC events

A.10.2 Weighing events

Many analyses require events to be weighted for cross section and luminosity, for trigger efficiencies, or with various scale factors. CutLang has a mechanism for applying constant event weights or event weights from functions, for which examples are shown below:

```
weight trigEff 0.95
weight ef2Weight myWeight({ELE_0}pT, {ELE_0}Eta)  # weight 2d
```

The first command sets the weight of the selected events to 0.95, i.e, if the number of selected events is 1000 in the beginning, now it will be counted as 950. The second command is a slightly more complicated example as it uses a table which defines the event weight according to two parameters: pT and η. The event weight is thus obtained from that table according to the attributes of the electron with the highest transverse momentum.

A.10.3 Saving events

In CutLang , it is possible to save the currently surviving events at any stage of the running algorithm. The events are saved into a ROOT [4] file using the command `save` followed by the user-given file name without the .root extension which is automatically added. It is possible to save multiple times in a single algorithm (region) or multiple algorithms. The events in the output file are saved in the native format of CutLang , known as the `lvl0` file. Therefore an example could be:

```
Save   preselects
```

A.11 Bins, counts and histograms

A.11.1 Bins

In analyses dealing with multiple bins for signal and/or background regions, CutLang provides a simple way for defining the selections for those bins. The binning of the results should happen as the very last stage of a selection by using the keyword `bin`. Either the variable and the bin boundaries should be explicitly listed, or multiple bins can be assigned to any variable or function using CutLang syntax. These two methods are not mutually exclusive and can define overlapping regions. It is to be noted that for the former, one defines two implicit bins: anything below the first value, and anything above the last value are also recorded separately.

Results from binning are both printed (depending on the switches in the initialization section of the ADL file) and recorded as a histogram in the output ROOT file. The examples below illustrate the utilization of the bin definition in an analysis algorithm:

```
bin MET 250 300 500 750 1000 # defining multiple bins simultaneously
bin Size(bjets) == 1  AND  HT [] 500 1000 # defining a single bin
bin Size(bjets) == 1  AND  HT [] 1000 1500 # defining a single bin
```

A.11.2 Counts

It is possible to register various signal, background or data counts of a region together with their associated errors. The method to achieve this task is to start the ADL file with the definitions of various count formats. Below are two such examples where for each format type, multiple processes with different names can also be defined.

```
countsformat results
    process est, "Total estimated BG", stat, syst
    process obs, "Observed data"

countsformat bgests
    process lostlep, "Lost lepton background", stat, syst
    process zinv, "Z --> vv background", stat, syst
    process qcd, "QCD background", stat, syst
```

A study described in an ADL file might use data counts or a background estimate or all of these for a statistical analysis. Therefore, the appropriate region has to contain the associated event counts and error information using the correct syntax. It should be consistent with the previous definitions starting with keyword `counts`. Here the counts of each process should be separated by a comma, and the errors can be specified either as symmetrical denoted with the `+-` sign or asymmetrical denoted with separate `+` and `-` signs. An example conforming to above definitions is given below.

```
counts results 230.0 + 16.0 - 10.0 + 10.0 - 12.0  , 224.0
counts bgests 105.0 +16.0 - 10.0 +-1.0 , 123.0 +-2.0 +-12.0 , 2.3 +-0.5 +-1.4
```

Once the analysis run is complete, the user finds in the output file a histogram for each of the defined processes with the name defined in the format commands. These histograms can be recalled and used later during the statistical analysis stage.

A.11.3 Histograms

CutLang allows defining 1D and 2D histograms for any event variable. The syntax for defining histograms follows closely the notation in ROOT. Any histogram should have a name, like `h1mReco`, and a list of parameters separated by commas. The explanation of the histogram contents should be given in quotation marks, e.g., `‘‘Z candidate mass (GeV)"`; the number of bins, lower and upper limits as numbers, e.g. `100, 0, 200`; and finally the quantity to histogram with the ADL notation, e.g. `{ELE_0 ELE_1}m`. A similar syntax is also used for the 2D histograms. The example below show definitions of 1D and 2D histograms:

```
region Wtopmass
    select ...
    select ...
    hmW,"W mass (GeV)", 70, 50, 150, mW
    hmTop,"Top mass (GeV)", 70, 0, 700, mTop
    hmTopmW,"Top and W mass correlation (GeV)", 50, 50, 150, 70, 0, 700, mW, mTop
```

Apart from the user-defined histograms, CutLang by default automatically fills and saves a cutflow efficiency histogram for each analysis region. In case binning exists, CutLang also saves a histogram with bin counts.

Figure 1 shows a snapshot of the `ROOT TBrowser`, with histograms in an output file listed, and one of the histograms displayed.

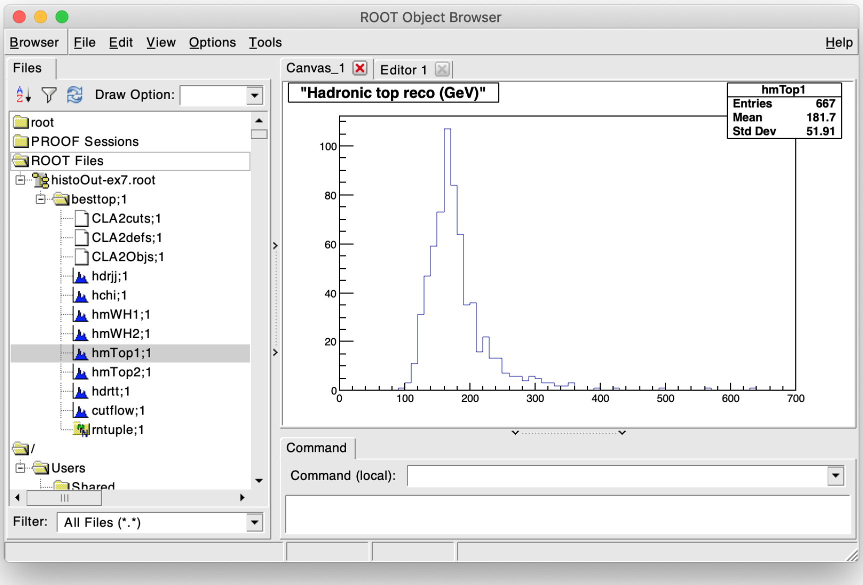

Figure 1: An example output from ROOT's TBrowser GUI showing histograms booked and filled by CutLang

A.12 Structure of a complete ADL file

To be run with CutLang , an ADL file should follow a definite structure order as described in Section ??. In this structure, there are mostly optional sections and one compulsory section. The structure order consists of initialization, count format, definitions, new objects, more definitions using new objects, yet newer objects, and event categorization commands. In this list only the event categorization commands are mandatory. The ADL file structure allows multiple concurrent commands to be executed. The details of the first and the last sections are covered next.

A.12.1 Initialization and information section

Some of the possible settings in the initialization section have already been discussed in Table ??. It is also possible to include, in this section, some information defining the work that is being done. The allowed keywords and their meaning is explained in the table below.

Table 12: Information keywords of CutLang

Keywords	Type	Explanation
`info`	ID	a name defining the work
`experiment`	ID	a name defining the experiment
`id`	`string`	any string defining the work
`title`	`string`	any string for the paper title
`publication`	`string`	any string, the publication information
`sqrtS`	`number`	a real number, the collider energy (GeV)
`lumi`	`number`	a real number, collected data (fb-1)
`arXiv`	`string`	any string containing the arxiv information
`hepdata`	`string`	any string containing the hepdata information
`doi`	`string`	any string containing the doi information

A.12.2 Regions and algorithms

CutLang can execute multiple commands in the event categorization section of the ADL file, meaning that the analyst can test multiple methods on the same events independently of each other during design, or work

with multiple signal and control regions. The set of commands to be executed for each independent method is called either an algorithm or a region, therefore the keyword to be used is `algo` or `algorithm` or `region` followed with a user selected name, such as:

```
region preselection
```

Moreover it is possible to define one (1) layer of dependency such that a region can be marked as dependent on another. In this case, the independent region's commands are executed first and the results are saved in a memory cache, and later the dependent region's commands are executed based on that cache. A typical case would be to create multiple signal regions based on a common preselection. This example is illustrated below. Note that the name of the independent region has been used in the dependent region's list of commands directly, without any preceding keywords.

```
region preselection
    select ....

region SRA
    preselection
    select ....

region SRB
    preselection
    select ....
```

B The CutLang framework

B.1 installation and compilation

The code for the CutLang framework can be found in

https://github.com/unelg/CutLang

The ROOT library from CERN should be pre-installed. After downloading the source code, the `make` command should be executed in the `CLA` subdirectory to compiles the whole program. Analyses in CutLang are run runs subdirectory using the script `CLA.sh` or `CLA.py`. This subdirectory contains several example files that demonstrate various aspects of ADL and CutLang . An analysis can be run using the command where the input ROOT file type can be : `LHCO FCC LVL0 DELPHES ATLASVLL ATLMIN ATLASOD CMSOD CMSNANO` . The `-i` or `--inifile` option is used for specifying the adl file.

B.2 External user functions

The addition of the new so called external user functions to the existing set of internal functions is partially automatized. The python helper script `insertExternalFunction.py` in the scripts directory is developed to accomplish this task. It accepts the name of the header file containing the new function as an argument. The automatization currently works with a template based setup, therefore only with certain type of functions. Currently the following input and return types for external functions can be used for building an external function into CutLang :

- receives a vector of TLorentzVectors and an int, returns a vector of TLorentzVector;
- receives a vector of TLorentzVectors, returns a double;
- receives a vector of TLorentzVectors and a TVector2, returns a double;
- receives a vector of TLorentzVectors and a TLorentzVector, returns a double;
- receives 3 TLorentzVectors, returns a double;

The external function must be declared and defined using C/C++ programming language in a header file before running the script. The script is run with the following command:

```
python insertExternalFunction.py -ext abc
```

where `abc` is name of the header file without `.h` extension. Once the helper script runs successfully, the CutLang binary has to be recompiled once to use the new function within an ADL file.

B.3 Incorporation of new input file types

This section describes how to build the interface between a new data file format represented as a flat ntuple and the standard types used by the *CutLang* interpreter. This is one aspect of the current version of *CutLang* that requires some coding expertise. *CutLang* uses ROOT's `MakeClass` for this purpose.

- Obtain a sample ROOT ntuple file containing the new data format and load into ROOT (e.g., using `TFile f("myfile.root")`)
- Call the ROOT `MakeClass` command on the relevant tree, specifying a class name

  ```
  tree->MakeClass("NewFormatName");
  ```

- Move the resulting header file (`NewFormatName.h`) into the `analysis_core` subdirectory, and is include it in the main code `CLA.Q`
- Move the resulting implementation macro (`NewFormatName.C`) into the `CutLang/CLA` directory, and include the following required headers in it:

  ```
  #include <NewFormatName.h>
  #include <TH2.h>
  #include <TStyle.h>
  #include <TCanvas.h>
  #include <signal.h>
  #include <TObject.h>
  #include <TBranchElement.h>

  #include "dbx_electron.h"
  #include "dbx_muon.h"
  #include "dbx_jet.h"
  #include "dbx_tau.h"
  #include "dbx_a.h"
  #include "DBXNtuple.h"
  #include "analysis_core.h"
  #include "AnalysisController.h"
  ```

- In the event loop, the input data must be transferred to the standard CutLang types, e.g., the electron, muon, photon and jet particle vectors, without forgetting any available event-wide information like RunNumber, EventNumber etc. An example conversion for the `LHCO` format is:

  ```
  TLorentzVector alv; dbxMuon *adbxm; vector<dbxMuon> muons;
  for (unsigned int i=0; i<Muon_; i++) {
    alv.SetPtEtaPhiM(Muon_PT[i], Muon_Eta[i], Muon_Phi[i], (105.658/1E3)); //in GeV
    adbxm= new dbxMuon(alv);
    adbxm->setCharge(Muon_Charge[i] );
    adbxm->setEtCone(Muon_ETiso[i] );
    adbxm->setPtCone(Muon_PTiso[i] );
    adbxm->setParticleIndx(i);
    muons.push_back(*adbxm);
    delete adbxm;
  }
  ```

- Modify the end of the .C file to be as follows:

  ```
  AnalysisObjects a0={muos_map, eles_map, taus_map, gams_map, jets_map, ljets_map,
                truth_map, combo_map, constits_map, met_map, anevt};

  aCtrl.RunTasks(a0);
  } // end of event loop
  aCtrl.Finalize();
  } // end of Loop function
  ```

- Modify the running script (CLA.sh or CLA.py) to incorporate the new file format.

References

[1] “PDG Particle Identification Numbers.” `https://pdg.lbl.gov/2013/pdgid/PDGIdentifiers.html`.

[2] P. D. Group, P. A. Zyla, et al., *Review of Particle Physics*, *Progress of Theoretical and Experimental Physics* **2020** (2020) [`https://academic.oup.com/ptep/article-pdf/2020/8/083C01/33653179/ptaa104.pdf`]. 083C01.

[3] M. Matsumoto and T. Nishimura, *Mersenne twister: a 623-dimensionally equidistributed uniform pseudo-random number generator*, *ACM Trans. Model. Comput. Simul.* **8** (1998) 3–30.

[4] R. Brun and F. Rademakers, *ROOT - An Object Oriented Data Analysis Framework*, *Nucl. Inst. and Meth. in Phys. Res. A* (1997) 81–86.
